# Gender perception of pareidolia faces in emergency department patients: the influence of physician gender

**DOI:** 10.3389/fnhum.2025.1628840

**Published:** 2025-12-19

**Authors:** Melih Çamcı, Gülsüm Akdeniz, Harun Demirci, Esra Demir Ünal, Nilgün Altuntaş

**Affiliations:** 1 Department of Emergency Medicine, Ankara Bilkent City Hospital, Ankara Yıldırım Beyazıt University, Ankara, Türkiye; 2 Department of Neuroscience, Institute of Health Sciences, Ankara Yıldırım Beyazıt University, Ankara, Türkiye; 3 Department of Biophysics, Faculty of Medicine, Ankara Yıldırım Beyazıt University, Ankara, Türkiye; 4 Department of Neurosurgery, Ankara Bilkent City Hospital, Ankara Yıldırım Beyazıt University, Ankara, Türkiye; 5 Department of Neurology, Ankara Bilkent City Hospital, Ankara Yıldırım Beyazıt University, Ankara, Türkiye; 6 Department of Neonatology, Ankara Bilkent City Hospital, Ankara Yıldırım Beyazıt University, Ankara, Türkiye

**Keywords:** emergency service, gender, face perception, female physician, pareidolia

## Abstract

**Background:**

The assessment of gender perception influenced by ambiguous facial cues in patients requiring emergency medical attention remains ambiguous. Pareidolia faces represent unconscious errors in facial recognition, wherein a wide array of visual attributes contribute to the interpretation of facial features. This study aims to explore the mechanisms underlying gender perception in individuals undergoing emergency medical treatment, employing an innovative digital pareidolia assessment to evaluate gender perception within the context of face pareidolia.

**Methods:**

Fifty adult patients treated by a female physician in the green triage zone participated in the study. Target images consisted of face pareidolia images, while non-target images were scrambled. All images were standardized for size, tone, and light intensity. Patients instructed the pareidolia images and were asked if they discerned a face; if they answered ‘No,’ the next image was shown. If they saw a face, they identified the associated gender. Their responses and reaction times were systematically recorded digitally.

**Results:**

Our findings revealed that, regardless of wait times, patients were significantly more likely to identify pareidolia faces as male rather than female, especially after being examined by a female physician. Additionally, male patients exhibited slightly longer reaction times than females when responding to pareidolia images.

**Conclusion:**

The outcomes of this investigation provide critical insights into the influence of pareidolia on gender perception of faces in the emergency department setting. It underscores the notion that gender biases, which arise from both biological and sociocultural factors, can affect the dynamics of patient-physician interactions.

## 1 Introduction

The demographic makeup of medical physicians has become a focal point of intensified scholarly discussion in recent years, reflecting substantial societal changes and the evolving landscape of the healthcare profession. As an increasing number of women enter the medical field and challenge entrenched stereotypes, it becomes imperative to comprehend the ramifications of gender diversity among physicians in fostering a more inclusive healthcare milieu. The growing representation of female physicians within the historically male-dominated medical domain has coincided with heightened inspection of patient satisfaction and the intricacies of doctor-patient interactions in contemporary discourse. Effective communication between physicians and patients is fundamental for augmenting patient satisfaction and ensuring adherence to prescribed treatment regimens (Clever et al., 2008; Gangopadhyay, 2024). The emergency department (ED) presents unique challenges to these interactions owing to its high-stakes and emotionally charged environment. In contrast to outpatient or clinic settings, where patients may have the opportunity to select their physician based on gender, the compulsive nature of care in the ED usually precludes such preferences (Duberstein et al., 2007; Nolen et al., 2016). Grasping how gender perceptions influence the patient-physician relationship in this context is vital, particularly as the prevalence of female physicians continues to escalate. Recognizing these biases is critical for ED practitioners to enhance communication, foster rapport, and secure improved patient outcomes (Nolen et al., 2016).

Gender perceptions significantly influence the dynamics of the patient-physician relationship, thereby affecting critical clinical outcomes such as treatment adherence, communication efficacy, and overall healthcare satisfaction. Specifically, existing research indicates that patients often harbor gender biases against female practitioners, which are frequently expressed through more informal or emotional qualitative feedback compared to evaluations of male counterparts (Gupta and Jordan, 2022). This subtle yet pervasive bias can undermine perceptions of professional competence, potentially affecting a patient’s willingness to communicate openly and consequently leading to issues with treatment adherence and satisfaction (Alhomayani et al., 2025). Furthermore, the relationship is complicated by explicit gender preferences among patients, particularly within culturally sensitive settings, where preferences for a same-gender physician are often rooted in deep-seated cultural and social norms (Alhomayani et al., 2025) demonstrably impacting patient comfort. Finally, the role of implicit bias, originating from either party, is critical, as the anticipation of bias can restrict vital clinical communication for example, prompting patients, particularly women, to avoid discussing certain sensitive topics resulting in suboptimal healthcare experiences and outcomes (Hernandez, 2023). Given that effective, empathetic communication is the cornerstone of successful patient engagement and improved outcomes (Campos et al., 2024). The investigation of underlying, potentially unconscious gender attribution biases, such as those revealed by face pareidolia, provides a necessary psychocognitive framework for understanding these complex clinical interactions.

The human visual system is highly optimized for the rapid detection of faces, a process for social survival. This specialization is so robust that it drives the phenomenon of pareidolia, wherein the brain swiftly interprets minimal visual cues in ambiguous, non-face stimuli as coherent facial structures (Gupta and Dobs, 2025). Pareidolia represents the neuroscientific phenomenon whereby individuals perceive familiar patterns, such as faces or objects, in random stimuli, often resulting in compelling interpretations and associations (Pavlova et al., 2016, 2020). Notably, face pareidolia activates brain regions linked to social attention, indicating that the human brain is innately predisposed to detect faces, even within ambiguous stimuli, thereby underscoring the importance of social perception in human cognition (Palmer and Clifford, 2020). The phenomenon is supported by a neural network comprising key components of the face processing network, notably the fusiform face area and the prefrontal cortex. These regions, in conjunction with the inferior and middle occipital cortices, are crucial for visual perception and the formation of perceptual inferences (Von Gal et al., 2023). Further research indicates the involvement of the posterior parietal cortex in the visual attention and perceptual processes associated with pareidolia (Von Gal et al., 2023) and suggests that stimulation of the right lateral occipito-temporal cortex and the left prefrontal cortex can enhance this phenomenon (Palmisano et al., 2023). Additionally, studies on cerebral blood flow in patients with dementia with Lewy bodies have demonstrated correlations between pareidolia and a complex network of regions, including the bilateral frontal lobes, the left cingulate cortex, and the left angular and supramarginal gyri (Nakata et al., 2022). These findings underscore the role of intricate brain network interactions in pareidolia, extending beyond primary visual processing regions (Palmisano et al., 2023). This phenomenon not only offers critical insights into facial sensitivity but also demonstrates that even stimuli devoid of actual facial components can communicate complex personal attributes, including emotional expressions, age, and gender (Alais et al., 2021). Empirical studies illustrate a marked advantage in recognizing happiness in female faces, wherein participants identify happiness more swiftly than anger or fear (Tay, 2015; Lipp and Taubert, 2024). Conversely, male faces are frequently associated with anger, which results in a perceptual advantage in recognizing this particular emotion (Guillin et al., 2023). Such gender-based perceptual biases commence early in development, influencing how children categorize faces and interpret social cues, potentially shaping long-term behaviors in diverse environments, including healthcare settings (Wardle et al., 2023).

The persistence of gender stereotypes, shaped by cultural traditions and social conditioning (Aliyeva, 2024), may play a crucial role in the cognitive mechanisms of pareidolia, where individuals assign gender-specific characteristics to ambiguous visual stimuli (Pavlova et al., 2016; Wardle et al., 2022). Moreover, even infants as young as 6 months are able to differentiate male from female faces, indicating that gender perception is a fundamental cognitive function influenced by biological factors (Kosakowski et al., 2022). This insight is crucial for understanding how gender bias affects healthcare, especially through face perceptions (Palmer and Clifford, 2020). Patients’ perceptions of physicians is strongly influenced by factors such as appearance, professionalism, and gender in clinical settings and gender stereotypes can lead to misinterpretations of physician’s intentions and capabilities, further complicating patient-physician dynamics (Palmer and Clifford, 2020; Chwe et al., 2024). These perceptions impact the trust and satisfaction levels of patients, underlining the importance of understanding how these biases affect patient-physician interactions and overall care quality (Kurihara et al., 2014). Additionally, gender stereotypes persistently shape the way faces are interpreted; studies show that ambiguous faces are more often categorized as male, reflecting deeply ingrained societal norms (Lipp and Taubert, 2024).

Currently, there is a lack of research specifically addressing patients’ perceptions of face gender, particularly within the context of emergency departments. This study aims to explore patterns of gender perception in patients undergoing emergency medical treatment, utilizing a novel digital pareidolia test (PT) to evaluate gender attribution in face pareidolia. Drawing upon existing literature and the cognitive male bias in face perception, we propose the hypothesis that patients in emergency departments will predominantly assign male gender to ambiguous face pareidolia stimuli, regardless of the gender of the attending physician.

## 2 Materials and methods

### 2.1 Study design

This study was designed as a cross-sectional, observational study and conducted in the Emergency Medicine Department of Ankara Bilkent City Hospital. Ethical approval was obtained from the Health Sciences Ethics Committee of Ankara Bilkent City Hospital (approval number: 2-24-466). The study was carried out in accordance with the Declaration of Helsinki, and digital informed consent was obtained from all participants. This study was conducted in accordance with the Sex and Gender Equity in Research (SAGER) guidelines to ensure the appropriate consideration of sex and gender aspects in research design, analysis, and interpretation.

### 2.2 Participants

**Figure 1:**
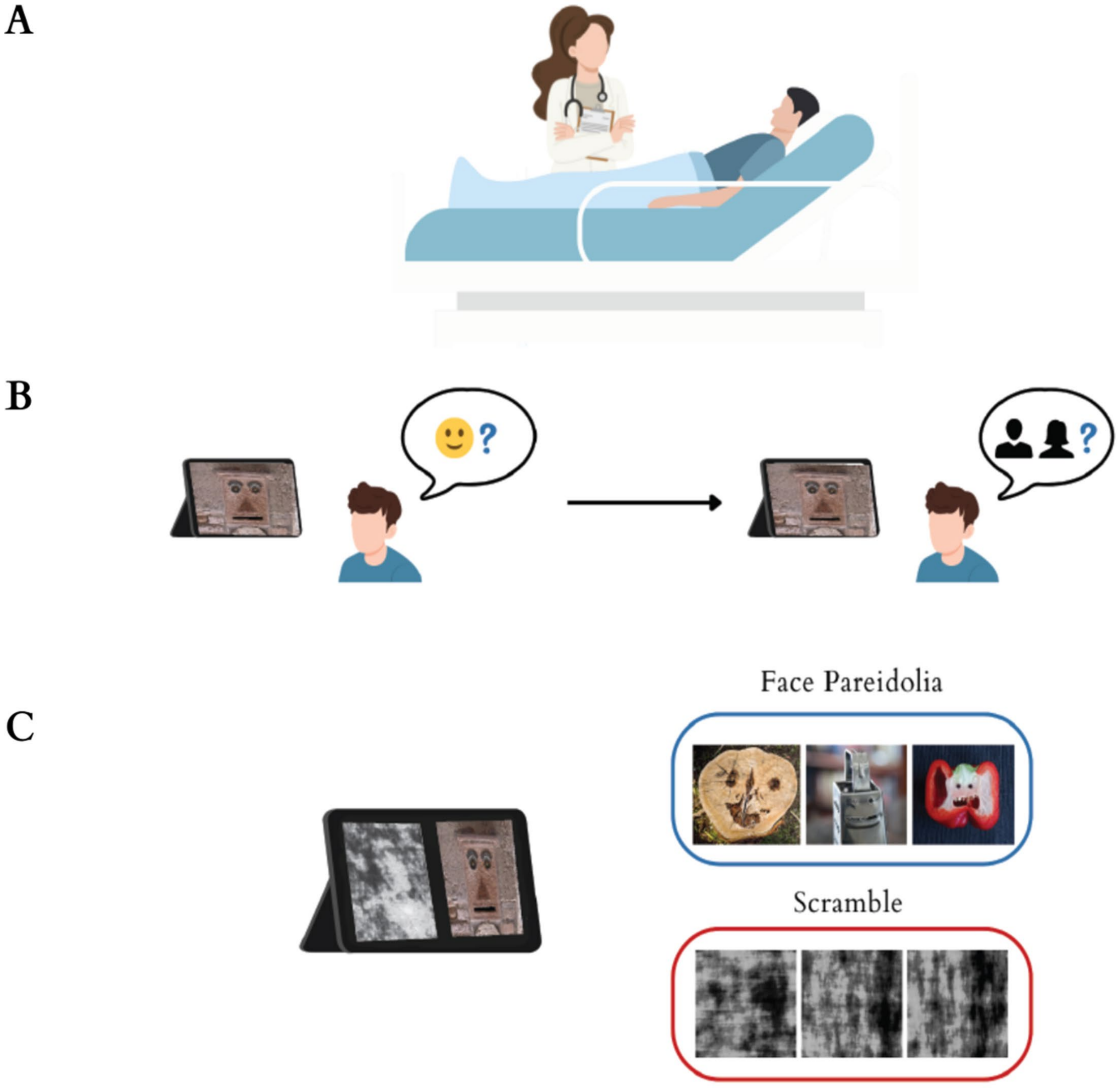
**(A)** Participants comprised patients who presented to the emergency department’s green zone with non-urgent medical complaints and were assessed, managed, and treated by a female physician. **(B)** Participants were presented with images and asked if they could perceive a face in the image. If they answered “yes,” they were further questioned about assessing the gender of the face. If they answered “no,” they moved on to the next image question. **(C)** Participants were presented with 10 images, consisting of face pareidolia images as target stimuli and scrambled images as non-target stimuli.

The study cohort was derived from the ED patient population who met the specified inclusion criteria. Inclusion criteria for participation were rigorously established as follows: (1) Adult patients presenting to the green triage zone. The green zone represents the lowest triage acuity category in the Emergency Severity Index system (Van Wegen et al., 2025), designated for patients with stable, non-urgent conditions who do not require immediate critical intervention and may be managed with observation or minor treatment; (2) aged 18 years or older; (3) These individuals must have presented with non-urgent medical complaints such as rash, chronic headache, cold symptoms; (4) they must have been treated by the designated female physician central to the study design; (5) reliably complete the digital PT. Conversely, patients meeting any of the following conditions were subject to exclusion: (1) Those presenting to the yellow or red triage zones due to the requirement for urgent or critical care; (2) individuals with known uncorrected visual impairments; (3) those with a confirmed history of neurological or psychiatric disorders; (4) patients presenting with acute intoxication or an altered mental status that could compromise reliable test performance; and (5) any patient exhibiting an inability to utilize digital devices necessary for the administration of the PT. Following the completion of their medical treatment, these patients were enrolled (Figure 1A). The study utilized a consecutive convenience sampling method. A total of 50 individuals meeting the inclusion criteria were selected for participation.

### 2.3 Digital pareidolia test (PT)

Participants were exposed to a total of 10 images which were selected from previously utilized sources (Akdeniz, 2024). To ensure the validity and reliability of the instrument, the digital pareidolia test was meticulously designed. For content validity, the pareidolia images were sourced from a validated repository (Akdeniz, 2018) where they had previously demonstrated high internal reliability (Cronbach’s alpha = 0.88) with a sample of 75 participants. The images were further standardized in terms of size, tone, and light intensity to ensure perceptual equivalence. Regarding reliability, a pilot test was conducted with 10 non-participant volunteers, yielding a Cronbach’s alpha of 0.82 for response consistency across images, indicating good internal reliability. Target images comprised face pareidolia images, whereas non-target images included scrambled images (Figure 1C). These images underwent digital processing for task presentation and were uploaded to the Qualtrics CoreXM platform (Qualtrics, Seattle, Washington, USA).

### 2.4 Procedure

The digital PT was administered in person immediately after the examination in a quiet consultation room of the emergency department, using a tablet computer with a 10.2-inch screen that was dedicated for the study. To minimize distractions, the test was conducted in a dimly lit environment. The testing conditions were kept consistent, with patients completing the task while seated at a dedicated station in the green triage area.

The test consisted of images presented in a randomized order to avoid sequence effects. For each image, participants were asked two sequential questions: (1) “Do you perceive a face in this image?” If the response was “No,” the system automatically advanced to the next image (Figure 1B). (2) If the response was “Yes,” participants were then asked: “Please identify the gender of the perceived face (Male/Female).” Responses were recorded via touchscreen buttons, with reaction times measured from image onset to response. Both responses and their precise reaction times were automatically recorded by the digital platform.

### 2.5 Statistical methods

Data analysis was performed using the Statistical Package for the Social Sciences (IBM SPSS v. 27). A significance level of *p* < 0.05 was considered statistically significant. The Shapiro–Wilk test was applied to assess the normality of the participants’ distribution. The results indicated a significant normality; therefore, parametric tests were employed in the analyses. Descriptive statistics, specifically median [min-max] values, were used to summarize the response variables and participants’ demographic information. For comparisons between groups and responses, the Paired Sample *t* Test and the Independent Sample *t* Test were applied. Correlation analysis was conducted to examine the relationships between reaction times and variables.

## 3 Results

**Figure 2:**
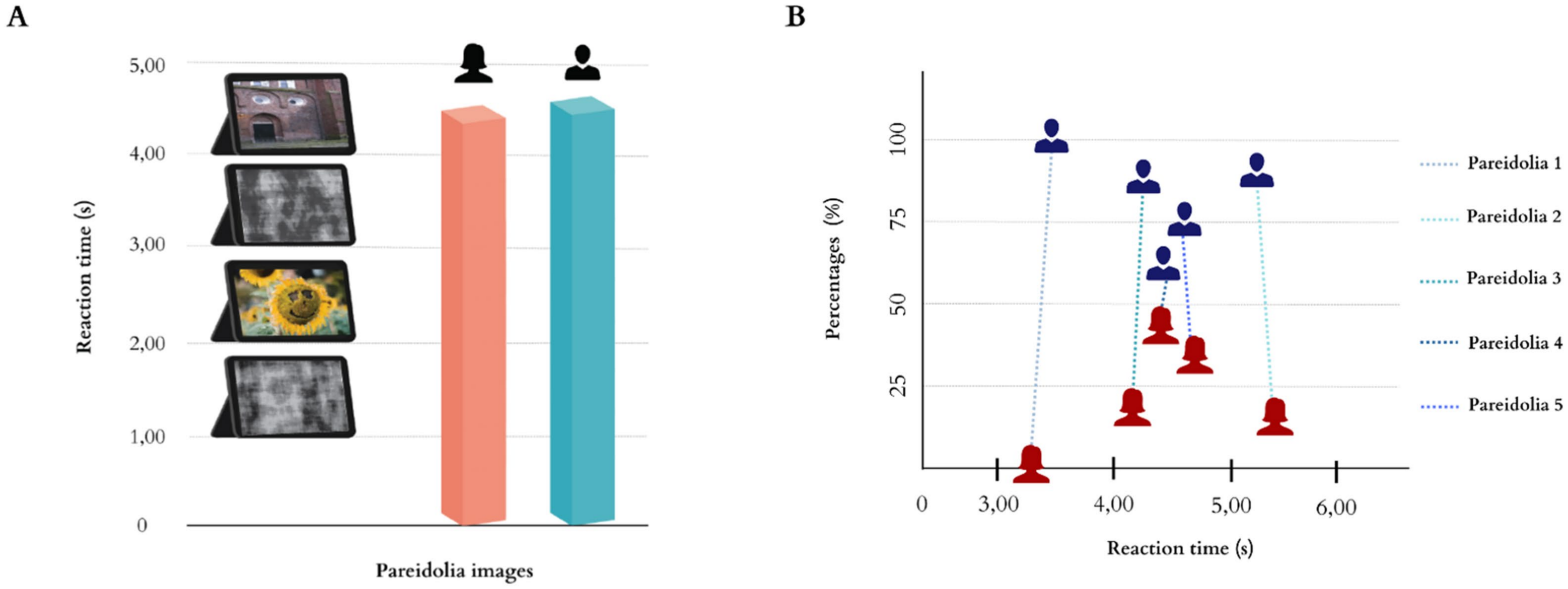
**(A)** Mean reaction time of the patients in response to the gender perception of pareidolia images. **(B)** Reaction time and percentage of each pareidolia image while patients respond to gender perception.

The study included 50 patients with ED (24 females, 26 males) and the majority of participants were in the 18–39 age group (*n* = 33), while the remaining 17 individuals were aged between 40 and 65. The demographic characteristics and referral diagnoses of the patients with ED are shown in Table 1. The reaction times of patients in response to face gender perception of the pareidolia images are depicted in Figure 2A. Male gender was more likely to be perceived (81%) than female gender (19%). Female patients with ED demonstrated a rate of identifying the female gender (20%) in contrast to the male gender (80%). Conversely, male patients with ED predominantly recognized the male gender (82%), while the identification of the female gender occurred less frequently (18%). Reaction time and percentage of each pareidolia image while patients respond to gender perception are illustrated in Figure 2B. An analysis of reaction times to pareidolia images stratified by gender indicated that patients exhibited significantly prolonged reaction times for male responses relative to female responses to pareidolia images (*p* = 0.000). We conducted an Independent Samples t-test to compare the mean perception time of face based on the pareidolia images by gender. The analysis revealed no statistically significant difference between female patients (Mean ± SD: 4.33 ± 0.99 s) and male patients (4.39 ± 1.23 s), *t*(48) = −0.76, *p* = 0.861 (Table 2). A Paired Samples *t*-test was performed to compare the mean reaction times for identifying faces as either female or male of perceived gender across the entire cohort. This analysis yielded a highly significant difference in reaction times, *t*(49) = −6.31, *p* < 0.001 (Table 3). Patients were significantly faster at perceiving a female face (1.44 ± 0.69 s) compared to a male face (3.25 ± 0.98 s), with a mean difference of −1.81 s. Table 4 presents the results of a Pearson Correlation analysis investigating the association between the mean reaction times for perceived female and perceived male responses to pareidolia images. The analysis identified a highly significant negative correlation between the reaction times for these two attribution types (*r* = −0.566, *p* < 0.01). This significant inverse relationship indicates that participants who were relatively quicker at attributing a female gender to the ambiguous stimuli tended to be slower when attributing a male gender, and vice versa.

**Table 1:** Patients’ demographic overview.

Variables	Total (*n*)
Gender	
Female / Male	24 / 26
Age	
18–39 / 40–65	33 / 17
Current status	
Single / Married	19 / 31
Stress Level	
None	10
Mild	14
Moderate	12
Much	14
Referral diagnoses*	
Diseases of the respiratory system	21
Diseases of the digestive system	10
Diseases of the genitourinary system	6
Diseases of the circulatory system	5
Endocrine, nutritional and metabolic diseases	3
Diseases of the skin and subcutaneous tissue	1
Other diseases	4

*International Classification of Diseases, ICD.

**Table 2:** Comparing the mean of perceiving face in the pareidolia images by gender of the patients.

Gender	Mean ± SD	Mean difference	*t*	*p*
Female	4.33 ± 0.99	−0.05	−0.76	0.861
Male	4.39 ± 1.23			

*t*, independent sample *t* test.

**Table 3:** Comparing the mean reaction time of male and female response of patients to pareidolia images by perceived gender.

Image	Perceived gender	Mean ± SD	Paired mean difference	*t*	*p*
Pareidolia	Female	1.44 ± 0.69	−1.81	−6.31	0.000*
	Male	3.25 ± 0.98			

*t*, paired sample *t* test.

**Table 4:** Pearson correlation analysis of mean reaction times for perceived female and perceived male gender attribution in pareidolia images.

Gender	Mean	SD	Perceived male	Perceived female
Female	1.44	0.69	−0.566**	1
Male	3.46	1.28	1	−0.566**

* *p* < 0.00; Pearson Correlation.

## 4 Discussion

The present study investigated the cognitive mechanisms of gender perception in the specific context of ED patients, who completed a digital face PT immediately following consultation with a female physician. Our principal finding indicates that patients, regardless of their gender or the immediate social cue presented by a female clinician, predominantly attributed a male gender to the ambiguous pareidolia stimuli, with over 80% of attributions being male. This outcome robustly supports the hypothesis that the male attribution bias originates from cognitive processes rather than perceptual ones (Wardle et al., 2022), affirming that this deeply ingrained cognitive heuristic is highly resistant to immediate external, situational influences. While our design does not permit direct causal inference regarding the impact of physician gender, the results shed light on potential biases in high-stress medical environments that warrant further comparative investigation. This consistent and pervasive male bias aligns with and supports the notion that the tendency to categorize face pareidolia as male has a cognitive rather than purely perceptual origin (Palmer and Clifford, 2020; Wardle et al., 2022). Three interconnected explanations account for this pattern: first, male-by-default categorization occurs because ambiguous stimuli lack minimal features for female identification (e.g., long hair, eyelashes), reverting to a sociocultural default (Wardle et al., 2022); second, without biological gender markers, the bias relies on heuristics rather than feature-based judgments, unlike real human faces (Bruce et al., 1993; Wild et al., 2000); third, it aligns with neurocognitive models of face processing, where the brain’s optimization for detection drives stereotypical male interpretations (Gupta and Dobs, 2025).

The most compelling aspect of our data is the persistence of the male bias despite the counter-cue of a single, highly salient female treating physician. The clinical encounter is an explicit social interaction involving a professional female authority figure, yet this context failed to significantly alter the implicit gender attribution measured via the pareidolia task. This suggests that the automatic sociocognitive processes governing face recognition operate independently of, and may override, immediate explicit social priming in stressful, high-demand cognitive environments such as the ED. This finding has critical implications for understanding the implicit challenges female professionals face; patients may be processing their professional roles through an unconscious cognitive filter that defaults to male, potentially impacting communication and perceived competence even when the explicit interaction is positive.

Perceptual and cognitive factors further influence gender perception in pareidolia. Perceptually, elements like image color, orientation, and associations can shape interpretations (Wardle et al., 2022). In our study, we minimized biases by using neutral, colorless images without gender-specific objects such as handbags or cakes and by maintaining neutral expressions. Stimuli such as mountains or coffee cups were selected for perceptual neutrality, yet the results suggest cognitive factors dominated, as the male bias persisted (Steephen et al., 2021; Wardle et al., 2022). Literature supports this cognitive origin, linking it to an adaptable face evaluation system shaped by social conditioning and development (Palmer and Clifford, 2020). This is critical because gender stereotypes persist through cultural traditions (Aliyeva, 2024), and preferences for facial femininity or masculinity vary by culture (Bjornsdottir et al., 2025). The cultural dimensions of Turkish society, marked by historical patriarchal structures and lower gender equality indices, likely exacerbate the male bias in pareidolia perception, reflecting entrenched social biases where ambiguity defaults to male categorizations (Mishra et al., 2019). This is consistent with cross-cultural evidence indicating stronger female face recognition in countries with low gender equality. A more profound examination of social bias reveals that such perceptions can perpetuate inequities in healthcare, underscoring why different outcomes would be expected in more gender-equitable cultural contexts.

The processing of male and female faces by the human brain is influenced by gender, affecting both response speed and neural reactions (O'Toole et al., 1998). Participants generally exhibited significantly longer reaction times when categorizing stimuli as male compared to female, with these measures showing a notable negative correlation. This pattern suggests quicker resolution for female attributions, potentially due to higher perceived femininity in certain stimuli or differential neural processing (O'Toole et al., 1998; Rekow et al., 2020). Although male participants demonstrated slightly longer overall reaction times than female participants, this difference did not achieve statistical significance. This tendency may arise from sex-specific neural mechanisms, where males might engage in more extensive deliberation when categorizing ambiguous stimuli, possibly reflecting a lower baseline sensitivity to social cues (Pavlova et al., 2020). Alternatively, it may reflect contextual factors in the emergency department, such as gender-related variability in stress responses. These findings align with broader evidence of sex differences in reaction times during face gender tasks (Bruce et al., 1993), supporting the notion that male participants may require additional cognitive resources to resolve pareidolia-related ambiguity. Previous research has indicated faster processing for highly masculine faces (Bruce et al., 1993), while infants show a preference for and greater ability to discriminate female faces (Quinn et al., 2002; Sugden, 2021). In adults, rapid recognition of male faces has been observed alongside stronger neural responses to female faces (O'Toole et al., 1998; Rekow et al., 2020; Schmuck et al., 2024), underscoring the complex and context-dependent dynamics of gender perception.

Demographic factors, such as age and educational level, may moderate the observed male bias in pareidolia gender perception. In our sample, younger participants could exhibit reduced bias due to ongoing cultural shifts in Türkiye toward greater gender equity, whereas older individuals might retain stronger stereotypes from traditional socialization. Similarly, higher education levels, though not evaluated in this study, have been associated with attenuated gender biases in face perception, particularly in contexts of evolving sociocultural norms (Mishra et al., 2019). Future studies should stratify analyses by these variables to assess their moderating effects, especially in rapidly changing societies like Türkiye, where historical patriarchal structures likely amplified the male bias by defaulting ambiguity resolution to masculine categorizations (Pavlova et al., 2020).

### 4.1 Limitation

The interpretation of our results should be considered in light of several methodological limitations. Firstly, the investigation was predicated upon a limited convenience sample, which inherently constrains both statistical robustness and the extent to which findings can be generalized to the broader ED patient demographic. Importantly, the study exclusively involved patients treated by a female physician and did not include a comparative control group, thereby precluding the possibility of drawing definitive inferences concerning the specific influence of physician gender or the context of the ED. Secondly, notwithstanding the focus on non-critical patients, lingering effects stemming from pain or stress intrinsic to the ED environment may have diminished attentional engagement, potentially exacerbating heuristic biases such as the male default in facial perception. Participants’ health conditions in the ED may have influenced results by diminishing attentional engagement, leading to reliance on heuristic biases such as the male default in face perception. Although we targeted non-critical patients to minimize this, residual effects from pain or stress could have extended reaction times or reinforced sociocultural stereotypes. Lastly, the research was conducted within a singular hospital in Türkiye, thus the results may be influenced by cultural particularities related to gender roles. Subsequent investigations should aim to mitigate these limitations by utilizing larger, multicenter cohorts and integrating both healthy control participants and a physician gender balanced control group. Comparative studies with healthy controls are needed to clarify whether these findings are ED-specific or generalizable.

## 5 Conclusion

Our results demonstrate a reliable preference for perceiving male faces in naturally occurring errors of face gender perception. This study provides valuable insights into how face gender perception, shaped by cognitive mechanisms such as pareidolia, manifests in the emergency department setting. The findings suggest that gender biases, both inherent and socially constructed, may influence patient-physicians interactions, potentially affecting clinical outcomes. The use of a digital pareidolia test revealed notable differences in how male and female patients perceive ambiguous face stimuli, highlighting the need for further research into the implications of these perceptual biases in clinical practice. Future studies with larger and more diverse samples, as well as a balanced representation of physician genders, are necessary to deepen our understanding of these dynamics and to explore the broader applicability of pareidolia as a tool for assessing cognitive bias in healthcare settings.

## Data Availability

The raw data supporting the conclusions of this article will be made available by the authors, without undue reservation.

## References

[ref1] Akdeniz G. (2018). A validity and reliability study of Pareidolia test. Ankara Med. J. 18, 375–381. doi: 10.17098/amj.461661

[ref2] Akdeniz G. (2024). Face-like pareidolia images are more difficult to detect than real faces in children with autism spectrum disorder. Adv. Clin. Exp. Med. 33, 13–19. doi: 10.17219/acem/162922, 37166015

[ref3] Alais D. Xu Y. Wardle S. G. Taubert J. (2021). A shared mechanism for facial expression in human faces and face pareidolia. Proc. Biol. Sci. 288:20210966. doi: 10.1098/rspb.2021.0966, 34229489 PMC8261219

[ref4] Alhomayani K. M. Bukhary H. A. Aljuaid F. I. Alotaibi T. A. Alqurashi F. S. Althobaiti K. N. . (2025). Gender preferences in healthcare: a study of Saudi patients' physician preferences. Patient Prefer. Adherence 19, 295–303. doi: 10.2147/PPA.S494766, 39926247 PMC11806673

[ref5] Aliyeva S. (2024). Mechanisms ensuring persistence of gender stereotypes. Sci. Work 18, 172–180. doi: 10.62706/bqiz.2024.v18i1.123

[ref6] Bjornsdottir R. T. Holzleitner I. J. Ishii K. (2025). Preferences for facial femininity/masculinity across culture and the sexual orientation spectrum. J. Exp. Psychol. Gen. 154, 1284–1302. doi: 10.1037/xge0001720, 39869693

[ref7] Bruce V. Burton A. M. Hanna E. Healey P. Mason O. Coombes A. . (1993). Sex discrimination: how do we tell the difference between male and female faces? Perception 22, 131–152. doi: 10.1068/p220131, 8474840

[ref8] Campos C. F. C. Olivo C. R. Martins M. A. Tempski P. Z. (2024). Physicians' attention to patients' communication cues can improve patient satisfaction with care and perception of physicians' empathy. Clinics 79:100377. doi: 10.1016/j.clinsp.2024.100377, 38703716 PMC11087704

[ref9] Chwe J. A. H. Vartiainen H. I. Freeman J. B. (2024). A multidimensional neural representation of face impressions. J. Neurosci. 44:e0542242024. doi: 10.1523/JNEUROSCI.0542-24.2024, 39134420 PMC11426373

[ref10] Clever S. L. Jin L. Levinson W. Meltzer D. O. (2008). Does doctor-patient communication affect patient satisfaction with hospital care? Results of an analysis with a novel instrumental variable. Health Serv. Res. 43, 1505–1519. doi: 10.1111/j.1475-6773.2008.00849.x, 18459954 PMC2653895

[ref11] Duberstein P. Meldrum S. Fiscella K. Shields C. G. Epstein R. M. (2007). Influences on patients' ratings of physicians: physicians demographics and personality. Patient Educ. Couns. 65, 270–274. doi: 10.1016/j.pec.2006.09.007, 17125958

[ref12] Gangopadhyay S. A. (2024). Review-based study on the importance of doctor-patient communication in healthcare. J. Med. Dent. Sci. Res. 11, 87–90. doi: 10.35629/076X-11098790

[ref13] Guillin A. Chaby L. Vergilino-Perez D. (2023). He must be mad; she might be sad: perceptual and decisional aspects of emotion recognition in ambiguous faces. Cognit. Emot. 37, 1376–1385. doi: 10.1080/02699931.2023.2258585, 37732611

[ref14] Gupta P. Dobs K. (2025). Human-like face pareidolia emerges in deep neural networks optimized for face and object recognition. PLoS Comput. Biol. 21:e1012751. doi: 10.1371/journal.pcbi.1012751, 39869654 PMC11790231

[ref15] Gupta S. Jordan K. (2022). Understanding gender bias toward physicians using online doctor reviews. Psychol. Lang. Commun. 26, 18–41. doi: 10.2478/plc-2022-0002

[ref16] Hernandez R. (2023). "It's always among us. I can't act like it's not": women college students' perceptions of physicians' implicit Bias. Health Commun. 38, 50–60. doi: 10.1080/10410236.2021.1932107, 34036850

[ref17] Kosakowski H. L. Cohen M. A. Takahashi A. Keil B. Kanwisher N. Saxe R. (2022). Selective responses to faces, scenes, and bodies in the ventral visual pathway of infants. Curr. Biol. 32, 265–274.e5. doi: 10.1016/j.cub.2021.10.064, 34784506 PMC8792213

[ref18] Kurihara H. Maeno T. Maeno T. (2014). Importance of physicians' attire: factors influencing the impression it makes on patients, a cross-sectional study. Asia Pac. Fam. Med. 13:2. doi: 10.1186/1447-056X-13-2, 24397871 PMC3890493

[ref19] Lipp O. V. Taubert J. (2024). The face pareidolia illusion drives a happy face advantage that is dependent on perceived gender. Emotion 24, 1781–1787. doi: 10.1037/emo0001346, 38842878

[ref20] Mishra M. V. Likitlersuang J. B Wilmer J. Cohan S. Germine L. DeGutis J. M. (2019). Gender differences in familiar face recognition and the influence of sociocultural gender inequality. Sci. Rep. 9:17884. doi: 10.1038/s41598-019-54074-5, 31784547 PMC6884510

[ref21] Nakata T. Shimada K. Iba A. Oda H. Terashima A. Koide Y. . (2022). Correlation between noise pareidolia test scores for visual hallucinations and regional cerebral blood flow in dementia with Lewy bodies. Ann. Nucl. Med. 36, 384–392. doi: 10.1007/s12149-022-01717-9, 35092551

[ref22] Nolen H. A. Moore J. X. Rodgers J. B. Wang H. E. Walter L. A. (2016). Patient preference for physician gender in the emergency department. Yale J. Biol. Med. 89, 131–142, 27354840 PMC4918861

[ref23] O'Toole A. J. Deffenbacher K. A. Valentin D. McKee K. Huff D. Abdi H. (1998). The perception of face gender: the role of stimulus structure in recognition and classification. Mem. Cogn. 26, 146–160. doi: 10.3758/bf03211378, 9519705

[ref24] Palmer C. J. Clifford C. W. G. (2020). Face pareidolia recruits mechanisms for detecting human social attention. Psychol. Sci. 31, 1001–1012. doi: 10.1177/0956797620924814, 32697673

[ref25] Palmisano A. Chiarantoni G. Bossi F. Conti A. D'Elia V. Tagliente S. . (2023). Face pareidolia is enhanced by 40 Hz transcranial alternating current stimulation (tACS) of the face perception network. Sci. Rep. 13:2035. doi: 10.1038/s41598-023-29124-8, 36739325 PMC9899232

[ref26] Pavlova M. A. Mayer A. Hösl F. Sokolov A. N. (2016). Faces on her and his mind: female and likable. PLoS One 11:e0157636. doi: 10.1371/journal.pone.0157636, 27352016 PMC4924832

[ref27] Pavlova M. A. Romagnano V. Fallgatter A. J. Sokolov A. N. (2020). Face pareidolia in the brain: impact of gender and orientation. PLoS One 15:e0244516. doi: 10.1371/journal.pone.0244516, 33382767 PMC7774913

[ref28] Quinn P. C. Yahr J. Kuhn A. Slater A. M. Pascalils O. (2002). Representation of the gender of human faces by infants: a preference for female. Perception 31, 1109–1121. doi: 10.1068/p3331, 12375875

[ref29] Rekow D. Baudouin J. Y. Rossion B. Leleu A. (2020). An ecological measure of rapid and automatic face-sex categorization. Cortex 127, 150–161. doi: 10.1016/j.cortex.2020.02.007, 32200287

[ref30] Schmuck J. Voltz E. Gibbons H. (2024). You're beautiful when you smile: event-related brain potential (ERP) evidence of early opposite-gender Bias in happy faces. Brain Sci. 14:739. doi: 10.3390/brainsci14080739, 39199434 PMC11353154

[ref31] Steephen J. E. Kummetha S. Obbineni S. C. Bapi R. S. (2021). Mood-congruent biases in facial emotion perception and their gender dependence. Int. J. Psychol. 56, 378–386. doi: 10.1002/ijop.12720, 33015843

[ref32] Sugden N. A. (2021). Learning from experience: Exposure to, attention to, discrimination of, and brain response to faces at 3, 6, and 9 months. Toronto, ON: Toronto Metropolitan University.

[ref33] Tay P. K. (2015). The adaptive value associated with expressing and perceiving angry-male and happy-female faces. Front. Psychol. 6:851. doi: 10.3389/fpsyg.2015.00851, 26157405 PMC4476135

[ref34] van Wegen M. E. Fransen L. F. C. Thijssen W. A. M. H. Alexandridis G. de Groot B. (2025). The association between urgency level and hospital admission, mortality and resource utilization in three emergency department triage systems: an observational multicenter study. Scand. J. Trauma Resusc. Emerg. Med. 33:72. doi: 10.1186/s13049-025-01392-5, 40312391 PMC12044865

[ref35] von Gal A. Boccia M. Nori R. Verde P. Giannini A. M. Piccardi L. (2023). Neural networks underlying visual illusions: an activation likelihood estimation meta-analysis. NeuroImage 279:120335. doi: 10.1016/j.neuroimage.2023.120335, 37591478

[ref36] Wardle S. G. Ewing L. Malcolm G. L. Paranjape S. Baker C. I. (2023). Children perceive illusory faces in objects as male more often than female. Cognition 235:105398. doi: 10.1016/j.cognition.2023.105398, 36791506 PMC10085858

[ref37] Wardle S. G. Paranjape S. Taubert J. Baker C. I. (2022). Illusory faces are more likely to be perceived as male than female. Proc. Natl. Acad. Sci. USA 119:e2117413119. doi: 10.1073/pnas.2117413119, 35074880 PMC8812520

[ref38] Wild H. A. Barrett S. E. Spence M. J. O'Toole A. J. Cheng Y. D. Brooke J. (2000). Recognition and sex categorization of adults' and children's faces: examining performance in the absence of sex-stereotyped cues. J. Exp. Child Psychol. 77, 269–291. doi: 10.1006/jecp.1999.2554, 11063629

